# A mathematical study for psoriasis transmission with immune-mediated time delays and optimal control strategies

**DOI:** 10.1371/journal.pone.0334101

**Published:** 2025-10-17

**Authors:** Xianbing Cao, Subhankar Kushary, Tushar Ghosh, Fahad Al Basir, Priti Kumar Roy

**Affiliations:** 1 School of Mathematics and Statistics, Beijing Technology and Business University, Beijing, China; 2 Department of Mathematics, Centre for Mathematical Biology and Ecology, Jadavpur University, Kolkata, India; 3 Department of Mathematics, Asansol Girls’ College, Asansol, West Bengal, India; Shanxi University, CHINA

## Abstract

In psoriasis, dendritic cells activate T cells, which then release excessive pro-inflammatory cytokines, leading to abnormal growth of keratinocytes in the epidermis. At the same time, anti-inflammatory cytokines attempt to restore balance. In reality, these immune processes are not immediate; they involve biological time gaps due to signal processing, cell communication, and cytokine feedback. Such immune-related delays may play a key role in triggering unstable or oscillatory behavior observed in psoriasis flare-ups. In this study, we present and analyze a mathematical model of psoriasis that explicitly includes two intracellular immune-mediated time delays to demonstrate their biological significance in disease progression. The model captures the interactions among T cells, dendritic cells, keratinocytes, and local mature stem cells. It features two cytokine-mediated feedback loops between T cells and dendritic cells, while stem cells attempt to regulate the immune response through anti-inflammatory signaling. A key challenge is identifying the critical time delays that modulate these interactions. To address this, we introduce two different delays in different interaction terms of the model system. We test the hypothesis that these delays can critically influence the onset and persistence of psoriatic pathology mathematically. Using stability analysis of the interior equilibrium, we determine parametric relations, their ranges, and delay thresholds that give rise to Hopf bifurcations, thereby linking delays to disease and deriving conditions of instability. Our analysis demonstrates that both immune-mediated delays critically influence system stability, with threshold values of $\tau_{1}^{*}$ and $\tau_{2}^{*}$ inducing oscillations through Hopf bifurcations. Further, we apply optimal control strategies on the delayed system using the effects of two biologic agents: TNF-*α* and IL-17 inhibitors. Incorporation of optimal controls effectively stabilizes the immune response. Numerical simulations support these analytical findings and show that biologic interventions can effectively reduce keratinocyte density. Inclusion of immune-related delays, based on both analytical and numerical results, provides a more realistic understanding of psoriasis dynamics and helps optimize therapeutic approaches for psoriasis management.

## 1 Introduction

Psoriasis is a chronic autoimmune skin disease that affects around 2–3% of the global population [1]. It is mainly caused by abnormal immune regulation and excessive production of pro-inflammatory cytokines, which lead to rapid growth of keratinocytes and the formation of thick, scaly skin patches [2,3]. The disease not only reduces the physical quality of life but also creates psychological and social stress for patients [4]. Environmental factors such as smoking and alcohol consumption further increase the severity of psoriasis [5].

The interaction between T cells and dendritic cells (DCs) is considered the central mechanism in the development of psoriasis. Dysfunctional DCs activate T cells by releasing cytokines such as TNF-*α* and IL-23, while activated T cells further stimulate DCs through feedback signals including TNF-*α* and IL-17 [6,7]. This creates a self-sustaining loop that amplifies inflammation and promotes abnormal keratinocyte proliferation [8]. Although mesenchymal stromal cells (MSCs) attempt to regulate this imbalance by controlling cytokine release, they often lose their protective role during psoriatic lesions [9]. Thus, psoriasis progression can be seen as the outcome of multiple interacting immune processes, sustained by feedback mechanisms.

Several researchers have studied psoriasis using mathematical modeling to capture such complex biological interactions. Oza et al. [10] examined convergence properties in cytokine-driven systems, Roy et al. [11] analyzed cytokine signaling pathways and biologic control strategies. Kushary et al. [12] recently proposed a mathematical model for psoriasis. Later, they studied the fractional form of this model using three operators, namely Caputo, Caputo–Fabrizio, and Atangana–Baleanu in the Caputo sense [13].

Delays naturally arise in immune systems because cytokine signaling, cellular activation, infiltration, and subsequent responses are not instantaneous. Inspired by the work of Das et al. [14], who highlighted the role of delays in immune system modeling, we extend our previously proposed psoriasis model [12] by investigating the influence of immune-mediated delays. In the context of psoriasis, such delays can significantly alter disease dynamics by destabilizing the system or inducing oscillatory immune responses. To explore this, we formulate a delay-based mathematical model in which intracellular time delays are incorporated into the separate interaction terms of T cells and DCs mediated through cytokine signaling. We analyze the impact of these delays on disease progression, perform delay-induced Hopf bifurcation analysis, and design optimal control strategies on the delayed model using biologic inhibitors to regulate the system. Numerical simulations are further carried out to validate the theoretical findings. The novelty of this study lies in demonstrating how immune-mediated delays affect the stability of the psoriasis model and how biologic control strategies can be optimized within this delay-induced framework. We expect that the results of this work will provide useful insights for designing improved control strategies for psoriasis.

## 2 The model introducing immune delays

In this section, we present a mathematical model for psoriasis in the presence of immune-mediated delays. In our earlier work, Kushary et al. [12] formulated a four-dimensional deterministic model based on ordinary differential equations (ODEs). The detailed assumptions underlying that model are given in the cited reference. The system considered the main cellular populations involved in psoriasis, namely T cells, dendritic cells, keratinocytes, and mesenchymal stem cells, under appropriate biological assumptions. The present study extends that earlier model by incorporating time delays.

The interactions between T cells and dendritic cells, mediated by pro-inflammatory cytokines, are not instantaneous. Anti-inflammatory cytokines attempt to counterbalance these signals, which leads to a lag in immune activation and inflammatory cell infiltration. To capture this effect, we introduce two distinct intracellular immune response delays into the previous model. These delays represent the time gap in the interaction between T cells and dendritic cells, and their subsequent influence on keratinocyte proliferation.

Based on these considerations, the delayed mathematical model is formulated as follows:

$$\frac{dT(t)}{dt}=\rho_{T}+\pi T(t)(1-\frac{T(t)}{T^{max}})-\frac{\mu_{1}T(t-\tau_{1})D(t-\tau_{1})}{1+\epsilon_{1}S(t-\tau_{1})}-\delta T(t)K(t)-\eta_{T}T(t),\;\frac{dD(t)}{dt}=\rho_{D}-\frac{\mu_{2}T(t-\tau_{2})D(t-\tau_{2})}{1+\epsilon_{2}S(t-\tau_{2})}-\eta_{D}D(t),\;\frac{dK(t)}{dt}=\rho_{K}+\frac{\mu_{1}T(t-\tau_{1})D(t-\tau_{1})}{1+\epsilon_{1}S(t-\tau_{1})}+\frac{\mu_{2}T(t-\tau_{2})D(t-\tau_{2})}{1+\epsilon_{2}S(t-\tau_{2})}-\eta_{K}K(t),\;\frac{dS(t)}{dt}=\rho_{S}-\eta_{S}S(t),$$ (1)

subject to the initial conditions:


$$T(\theta )>0,\;D(\theta )>0,\;K(\theta )>0,\;S(\theta )>0,\text{where}\;\theta \in [-\tau ,0],\tau =\max\{\tau_{1},\tau_{2}\}.$$

**Fig 1:**
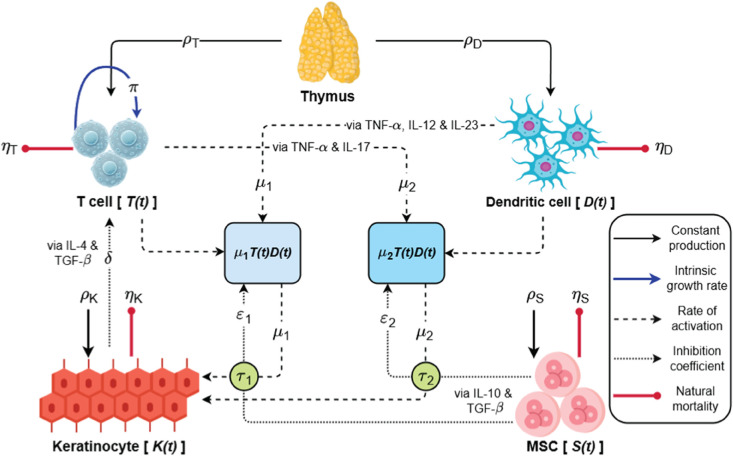
Schematic representation of model populations and their interrelationships mediated by cytokine signaling.

Here, *T*(*t*), *D*(*t*), *K*(*t*), and *S*(*t*) denote the population densities of T cells, dendritic cells, keratinocytes, and mesenchymal stem cells (MSCs), respectively, at time *t*. The interaction network among these cell populations is shown in the schematic diagram (Fig 1), and the model parameters are described in detail in Table 1. The parameter *τ* represents the time delay, measured in days.

**Table 1:** Parameter values used in the numerical simulations for the entire model system. Several parameter ranges were obtained from previous studies [12,24,25]. Due to the lack of sufficient primary data related to immune-mediated delay factors, some parameter values were chosen to ensure the biological plausibility of the model behavior.

Parameter	Definition	Value (Unit)
$\rho_{T}$	Constant accumulation rate of T cells	25 mm^−3^*d*^−1^
$\rho_{D}$	Constant accumulation rate of dendritic cells	25 mm^−3^*d*^−1^
$\rho_{K}$	Constant accumulation rate of keratinocytes	5 mm^−3^*d*^−1^
$\rho_{S}$	Constant production of all mature stem cells	0.5 mm^−3^*d*^−1^
*π*	Logistic growth rate T cells	0.003 *d*^−1^
$T^{\max}$	Maximum carrying capacity of T cells	300 mm^−3^
$\mu_{1}$	Rate of activation of T cells via dendritic cells	0.03 mm^3^*d*^−1^
$\mu_{2}$	Rate of activation of dendritic cells via T cells	0.03 mm^3^*d*^−1^
$\epsilon_{1}$	Inhibition scaling coefficient of MSCs on DC activation	0.05 mm^3^
$\epsilon_{2}$	Inhibition scaling coefficient of MSCs on T cell activation	0.05 mm^3^
*δ*	Rate of inhibition of T cells hyper-activity via keratinocyte	0.003 mm^3^*d*^−1^
$\eta_{T}$	Natural mortality rate of T cells	0.1 *d*^−1^
$\eta_{D}$	Natural mortality rate of dendritic cells	0.16 *d*^−1^
$\eta_{K}$	Natural mortality rate of Keratinocyte	0.25 *d*^−1^
$\eta_{S}$	Natural mortality rate of mature stem cells	0.3 *d*^−1^

Let $C=C([-\tau ,0],{\mathbb{R}}_{+}^{4})$ be the Banach space of all continuous functions $\varphi :[-\tau ,0]\to {\mathbb{R}}_{+}^{4}$, equipped with the usual supremum norm defined by:


$$||\varphi ||=\underset{-\tau <\theta <0}{\sup}\{|\varphi_{1}(\theta )|,|\varphi_{2}(\theta )|,|\varphi_{3}(\theta )|,|\varphi_{4}(\theta )|\},$$

where $\varphi =(\varphi_{1},\varphi_{2},\varphi_{3},\varphi_{4})$ and $T(\theta )=\varphi_{1}(\theta )$, $D(\theta )=\varphi_{2}(\theta )$, $K(\theta )=\varphi_{3}(\theta )$, $S(\theta )=\varphi_{4}(\theta )$ with θ∈[−τ,0]. To make the model feasible from the biological point of view, initial functions are assumed to be $\varphi_{i}(\theta )\geq$ 0 for θ∈[−τ,0] and $\varphi_{i}(0)>0$ for i=1,2,3,4. Using the fundamental theory of functional differential equations [15], we can guarantee the uniqueness of the solutions of the system (1) with the initial conditions mentioned above.

## 3 Mathematical properties of the delayed model

### 3.1 Positivity and boundedness

Positivity and boundedness in a mathematical model are essential to establish well-posedness. It provides a reliable basis for the prediction of the system and gives meaningful insight into the outcome of the model. In this subsection, we analyze the positivity and boundedness of the delay-induced system (1) through the following theorem.

**Theorem 1.** *Consider system (1) with initial history* $X(\theta )=(\varphi_{1}(\theta ),\varphi_{2}(\theta ),\varphi_{3}(\theta ),\varphi_{4}(\theta ))\in C([-\tau ,0],{\mathbb{R}}_{+}^{4})$ *such that* $\varphi_{i}(0)>0$ *for* i=1,2,3,4. *Then:*

1. *Every solution remains positive for all* t≥0*, i.e.,* $X(t)\in {\mathbb{R}}_{+}^{4}$*. Hence,* ${\mathbb{R}}_{+}^{4}$ *is a positively invariant region for* (1).
2. *Every positive solution is bounded and ultimately enters the domain of attraction* $\Delta \subseteq {\mathbb{R}}_{+}^{4}$, *where*
  $$\Delta =\{(T,D,K,S)\in {\mathbb{R}}_{+}^{4}:\;0<T+D+K\leq \frac{4\rho +\pi T^{\max}}{4\eta },\;\;0<S\leq \frac{\rho_{S}}{\eta_{S}}\},$$
  *with* $\rho =\rho_{T}+\rho_{D}+\rho_{K}$ *and* $\eta =\min\{\eta_{T},\eta_{D},\eta_{K}\}$.

**Proof:** Let $X(t)={\left(T(t),D(t),K(t),S(t)\right)}^{⊤}$ and


$$F(X)=(\begin{array}{c} \rho_{T}+\pi T(t)(1-\frac{T(t)}{T^{\max}})-\frac{\mu_{1}T(t-\tau_{1})D(t-\tau_{1})}{1+\epsilon_{1}S(t-\tau_{1})}-\delta T(t)K(t)-\eta_{T}T(t)\\ \rho_{D}-\frac{\mu_{2}T(t-\tau_{2})D(t-\tau_{2})}{1+\epsilon_{2}S(t-\tau_{2})}-\eta_{D}D(t)\\ \rho_{K}+\frac{\mu_{1}T(t-\tau_{1})D(t-\tau_{1})}{1+\epsilon_{1}S(t-\tau_{1})}+\frac{\mu_{2}T(t-\tau_{2})D(t-\tau_{2})}{1+\epsilon_{2}S(t-\tau_{2})}-\eta_{K}K(t)\\ \rho_{S}-\eta_{S}S(t) \end{array}).$$

Then system (1) can be written as

$$\frac{dX}{dt}=F(X),\;X(\theta )\in C([-\tau ,0],{\mathbb{R}}_{+}^{4}).$$ (2)

*1.* Let $x_{1}=T,\;x_{2}=D,\;x_{3}=K,\;x_{4}=S$. From the form of *F* it follows that


$$F_{i}(X){|}_{x_{i}=0,\;X\in {\mathbb{R}}_{+}^{4}}\;\geq \;0,\;i=1,2,3,4.$$

By Lemma 2 of Yang et al. [16] and Theorem 1.1 of Bodnar et al. [17], any solution *X*(*t*) of (2) with $X(\theta )\in C([-\tau ,0],{\mathbb{R}}_{+}^{4})$ satisfies $X(t)\in {\mathbb{R}}_{+}^{4}$ for all t≥0. Therefore, ${\mathbb{R}}_{+}^{4}$ is a positively invariant region for (1).

*2.* Adding the first three equations of (1) gives


$$\frac{d}{dt}(T+D+K)=\rho +\pi T(1-\frac{T}{T^{\max}})-\eta_{T}T-\eta_{D}D-\eta_{K}K\;\leq \;\rho +\pi T(1-\frac{T}{T^{\max}})-\eta (T+D+K),$$

where $\rho =\rho_{T}+\rho_{D}+\rho_{K}$ and $\eta =\min\{\eta_{T},\eta_{D},\eta_{K}\}$. The quadratic term satisfies $\pi T\left(1-\frac{T}{T^{\max}}\right)\leq \frac{\pi T^{\max}}{4}$. Hence,


$$\frac{d}{dt}(T+D+K)\;\leq \;(\rho +\frac{\pi T^{\max}}{4})-\eta \;(T+D+K).$$

By the comparison principle [18], for *t* > 0,


$$T(t)+D(t)+K(t)\;\leq \;\frac{4\rho +\pi T^{\max}}{4\eta }(1-e^{-\eta t})+(T(0)+D(0)+K(0))e^{-\eta t},$$

so for large *t*,


$$T(t)+D(t)+K(t)\;\leq \;\frac{4\rho +\pi T^{\max}}{4\eta }.$$

For the fourth equation we have the explicit solution


$$S(t)=\frac{\rho_{S}}{\eta_{S}}(1-e^{-\eta_{S}t})+S(0)e^{-\eta_{S}t}\;\;⟹\;\;S(t)\to \frac{\rho_{S}}{\eta_{S}}\;\text{as }t\to \infty .$$

Therefore, every positive solution ultimately enters and remains in


$$\Delta =\{(T,D,K,S)\in {\mathbb{R}}_{+}^{4}:\;0<T+D+K\leq \frac{4\rho +\pi T^{\max}}{4\eta },\;\;0<S\leq \frac{\rho_{S}}{\eta_{S}}\},$$

which satisfies $\Delta \subseteq {\mathbb{R}}_{+}^{4}$.

### 3.2 Feasibility of positive equilibrium

To determine the interior equilibrium point *E* (*T* , *D* , *K* , *S* ) of the delay-induced system, we set all the equations of the system (1) to zero and solve for the state variables under the conditions *T*  > 0, *D*  > 0, *K*  > 0, and *S*  > 0. From the fourth equation of the system (1), we get $S^{*}=\rho_{S}/\eta_{S}.$ Using the value of *S* , we introduce two constants, $\xi_{1}$ and $\xi_{2}$, to simplify the computations. These constants are defined as follows

$$\xi_{1}=\frac{\mu_{1}}{1+\epsilon_{1}S^{*}}\;\text{and}\;\xi_{2}=\frac{\mu_{2}}{1+\epsilon_{2}S^{*}}.$$ (3)

Subsequently, we determined the values of *D*  and *K*  in terms of *T*  using the aforementioned constants and by setting the second equation of the system (1) to zero. These values are given by

$$D^{*}=\frac{\rho_{D}}{\eta_{D}+\xi_{2}T^{*}}\;\;\text{and}\;\;K^{*}=\frac{\rho_{K}}{\eta_{K}}+\frac{\rho_{D}(\xi_{1}+\xi_{2})T^{*}}{\eta_{K}(\eta_{D}+\xi_{2}T^{*})}.$$ (4)

By setting the first equation of the system (1) to zero and substituting the obtained values of the state variables into this equation, we derive a cubic equation in terms of *T* .

$$T^{*3}+P_{1}T^{*2}+P_{2}T^{*}+P_{3}=0,$$ (5)

$$\text{where}\;\;P_{1}=\frac{1}{\pi \eta_{K}\xi_{2}}[\pi \eta_{K}\eta_{D}+\{(\delta \rho_{K}+\eta_{T}\eta_{K}-\pi \eta_{K})\xi_{2}+\rho_{D}\delta (\xi_{1}+\xi_{2})\}T^{\max}],\;P_{2}=\frac{1}{\pi \eta_{K}\xi_{2}}[\delta \rho_{K}\eta_{D}-\pi \eta_{D}\eta_{K}+\eta_{T}\eta_{D}\eta_{K}+\rho_{D}\eta_{K}\xi_{1}-\rho_{T}\eta_{K}\xi_{2}]T^{\max},\;P_{3}=-\frac{\rho_{T}\eta_{D}T^{\max}}{\pi \xi_{2}}.$$

Using the parameter values listed in Table 1, we observe that *P*_1_>0 is positive, while *P*_2_<0 and *P*_3_<0. According to Descartes’ rule of signs [19], the system (5) has exactly one positive root. By substituting the value of *T*  into (4), we can determine the interior equilibrium point *E* (*T* , *D* , *K* , *S* ).

### 3.3 Stability and Hopf-bifurcation analysis

The non-delayed system admits a unique positive interior equilibrium point, since the extinction of any population is biologically unrealistic. Without loss of generality, we denote this equilibrium as *E* (*T* , *D* , *K* , *S* ). The exact values of the state variables at *E*  were determined earlier in our previous work by Kushary et al. [12].

In this subsection, we study the local stability of the delay-induced system (1) in a neighborhood of the interior equilibrium point *E* .

Let $\hat{T}(t)=T(t)-T^{*},\hat{D}(t)=D(t)-D^{*},\hat{K}(t)=K(t)-K^{*},$ and $\hat{S}(t)=S(t)-S^{*}$ represent the perturbed variables near the equilibrium point *E* . Linearizing the delay-induced system (1) about *E* , we derive the following system

$$\frac{dY(t)}{dt}=J(0)Y(t)+J(\tau_{1})Y(t-\tau_{1})+J(\tau_{2})Y(t-\tau_{2}),$$ (6)

where $Y(t)=[\hat{T}(t),\hat{D}(t),\hat{K}(t),\hat{S}(t){]}^{T}$ and $J(0),J(\tau_{1})$, and $J(\tau_{2})$ represent the 4 × 4 Jacobian matrices of the system evaluated at *E*  for the current state, the delayed state with delay $\tau_{1}$, and the delayed state with delay $\tau_{2}$, respectively. Their explicit forms are

$$J(0)=(\begin{array}{cccc} -(\frac{\rho_{T}}{T^{*}}+\frac{\pi T^{*}}{Tmax}) & 0 & -\delta T^{*} & 0\\ 0 & -\eta_{D} & 0 & 0\\ 0 & 0 & -\eta_{K} & 0\\ 0 & 0 & 0 & -\eta_{S} \end{array}),\;J(\tau_{1})=(\begin{array}{cccc} -\mu_{1}D^{*} & -\mu_{1}T^{*} & 0 & \frac{\xi_{1}^{2}\epsilon_{1}T^{*}D^{*}}{\mu_{1}}\\ 0 & 0 & 0 & 0\\ \mu_{1}D^{*} & \mu_{1}T^{*} & 0 & -\frac{\xi_{1}^{2}\epsilon_{1}T^{*}D^{*}}{\mu_{1}}\\ 0 & 0 & 0 & 0 \end{array}),\;\text{and}\;J(\tau_{2})=(\begin{array}{cccc} 0 & 0 & 0 & 0\\ -\mu_{2}D^{*} & -\mu_{2}T^{*} & 0 & \frac{\xi_{2}^{2}\epsilon_{2}T^{*}D^{*}}{\mu_{2}}\\ \mu_{2}D^{*} & \mu_{2}T^{*} & 0 & -\frac{\xi_{2}^{2}\epsilon_{2}T^{*}D^{*}}{\mu_{2}}\\ 0 & 0 & 0 & 0 \end{array}).$$ (7)

Now, the characteristic equation of the system (1) is given by

$$|\lambda I-J(0)-e^{-\lambda \tau_{1}}J(\tau_{1})-e^{-\lambda \tau_{2}}J(\tau_{2})|=0,\;∴\;(\lambda +\eta_{S})[(\lambda^{3}+A_{1}\lambda^{2}+A_{2}\lambda +A_{3})+e^{-\lambda \tau_{1}}(B_{1}\lambda^{2}+B_{2}\lambda +B_{3})\;\;\;\;+e^{-\lambda \tau_{2}}(C_{1}\lambda^{2}+C_{2}\lambda +C_{3})]=0,$$ (8)

where the coefficients are


$$A_{1}=\frac{\rho_{T}}{T^{*}}-\xi_{1}D^{*}+\frac{\pi T^{*}}{T^{max}}+\eta_{D}+\eta_{K},\;A_{2}=(\frac{\rho_{T}}{T^{*}}-\xi_{1}D^{*}+\frac{\pi T^{*}}{T^{max}})(\eta_{D}+\eta_{K})+\eta_{D}\eta_{K},$$

$$A_{3}=(\frac{\rho_{T}}{T^{*}}-\xi_{1}D^{*}+\frac{\pi T^{*}}{T^{max}})(\eta_{D}+\eta_{K})\eta_{D}\eta_{K},\;B_{1}=\xi_{1}D^{*},$$

$$B_{2}=\delta \xi_{1}T^{*}D^{*}+\xi_{1}D^{*}(\eta_{D}+\eta_{K}),\;B_{3}=\eta_{D}\xi_{1}D^{*}(\delta T^{*}+\eta_{K}),\;C_{1}=\xi_{2}T^{*},$$

$$C_{2}=\delta \xi_{2}T^{*}D^{*}-\xi_{2}T^{*}(-\frac{\rho_{T}}{T^{*}}+\xi_{1}D^{*}-\frac{\pi T^{*}}{T^{max}}-\eta_{K}),$$

$$C_{3}=\delta \eta_{D}\xi_{2}T^{*}D^{*}+\eta_{K}\xi_{2}T^{*}(\frac{\rho_{T}}{T^{*}}-\xi_{1}D^{*}+\frac{\pi T^{*}}{T^{max}}).$$

The interior steady state *E*  is said to be locally asymptotically stable (LAS) if all roots *λ* of the characteristic Eq (8) are negative or possess negative real parts. The following cases will now be analyzed in detail.

**Case - I ($\tau_{1}$ = $\tau_{2}$ = 0)**

In this case, the characteristic Eq (8) simplifies to the following form

(λ+ηS)[λ3+A1λ2+A2λ+A3]=0,whereA1=A1+B1+C1, A2=A2+B2+C2,andA3=A3+B3+C3. (9)

One of the eigenvalues is $\lambda =-\eta_{S}<0$. Thus, the interior equilibrium *E*  is LAS in the absence of delays if following conditions are satisfied


A1>0, A3>0, and A1A2−A3>0.

**Case - II ($\tau_{1}>0$ and $\tau_{2}$ = 0)**

For this case, the characteristic Eq (8) takes the form

$$(\lambda +\eta_{S})[(\lambda^{3}+M_{1}\lambda^{2}+M_{2}\lambda +M_{3})+e^{-\lambda \tau_{1}}(B_{1}\lambda^{2}+B_{2}\lambda +B_{3})]=0,\;\text{where}\;\;M_{1}=A_{1}+C_{1},M_{2}=A_{2}+C_{2},\;\text{and}\;M_{3}=A_{3}+C_{3}.$$ (10)

The root $\lambda =-\eta_{S}$ is negative. Thus, we are only interested in analyzing the following equation

$$(\lambda^{3}+M_{1}\lambda^{2}+M_{2}\lambda +M_{3})+e^{-\lambda \tau_{1}}(B_{1}\lambda^{2}+B_{2}\lambda +B_{3})=0.$$ (11)

Since, the characteristic Eq (11) is transcendental in *λ*, the stability of the system (1) cannot be analyzed using the classical Routh-Hurwitz criteria [20]. The Eq (11) admits purely imaginary solutions as a necessary condition for a stability change at the interior equilibrium *E* . Let $\lambda =i\omega (\tau_{1})$ be a root of Eq (11). Substituting λ=iω into Eq (11) and separating the real and imaginary components, we obtain

$$M_{1}\omega^{2}-M_{3}=(B_{3}-B_{1}\omega^{2})\cos\omega \tau_{1}+B_{2}\omega \sin\omega \tau_{1},\;M_{2}\omega -\omega^{2}=(B_{3}-B_{1}\omega^{2})\sin\omega \tau_{1}+B_{2}\omega \cos\omega \tau_{1}.$$ (12)

Squaring and adding the above two equations and substituting $\omega^{2}=\sigma$, we get the following equation

$$\Psi (\sigma )\equiv \sigma^{3}+R_{1}\sigma^{2}+R_{2}\sigma +R_{3}=0,\;\text{where}\;\;R_{1}=M_{1}^{2}-2M_{2}-B_{1}^{2},R_{2}=M_{2}^{2}-2M_{1}M_{3}+2B_{1}B_{3}-B_{2}^{2},\;\text{and}\;R_{3}=M_{3}^{2}-B_{3}^{2}.$$ (13)

The Eq (11) does not admit purely imaginary roots if the Eq (13) satisfies the Routh-Hurwitz’s criteria [20]. The results are summarized in the following proposition.

**Proposition 1.** The interior equilibrium *E*  is LAS for all $\tau_{1}>0$ if the following conditions are hold


$$R_{1}>0,\;R_{3}>0,\;\text{and}\;\;R_{1}R_{2}-R_{3}>0.$$

If *R*_3_<0, the Eq (13) has at least one positive root. Let $\sigma =\omega^{2}$ denote the smallest positive root. In this case, the characteristic Eq (11) will have purely imaginary roots, says (±iω). We now determine the critical value of $\tau_{1}$, denoted as $\tau_{1}^{*}$ that corresponds to the point at which the stability of the interior equilibrium *E*  changes. The critical value $\tau_{1}^{*}$ is determined from the system of Eq (12) to analyze the conditions under which the system transitions from stability to instability as $\tau_{1}$ increases, as follows

$$\tau_{1}^{n}=\frac{1}{\omega }\cos^{-1}[\frac{(B_{2}-M_{1}B_{1})\omega^{4}+(M_{1}B_{3}+M_{3}B_{1}-M_{2}B_{2})\omega^{2}-M_{3}B_{3}}{B_{1}^{2}\omega^{4}+B_{2}+B_{3}^{2}}]+\frac{2n\pi }{\omega },\;\forall n\in \mathbb{N}\cup \{0\}.$$ (14)

Let us assume that for $\omega_{1}=\omega (\tau_{1})$, $\tau_{1}^{*}=\min_{n\geq 0}\{\tau_{1}^{n}\}$. Based on this assumption and summarizing the above discussions, we have derived the following theorem.

**Theorem 2.** *If R*_3_<0*, according to Butler lemma [21] the interior equilibrium E*  *is LAS when* $\tau_{1}<\tau_{1}^{*}$ *and becomes unstable when* $\tau_{1}>\tau_{1}^{*}$, where

$$\tau_{1}^{*}=\frac{1}{\omega_{1}}\arccos[\frac{(B_{2}-M_{1}B_{1})\omega_{1}^{4}+(M_{1}B_{3}+M_{3}B_{1}-M_{2}B_{2})\omega_{1}^{2}-M_{3}B_{3}}{B_{1}^{2}\omega_{1}^{4}+B_{2}+B_{3}^{2}}].$$ (15)

*Furthermore, when* $\tau_{1}=\tau_{1}^{*}$, a Hopf bifurcation [22] occurs, meaning that a family of periodic solutions of the system (1*) bifurcates as* $\tau_{1}$ *crosses the critical value* $\tau_{1}^{*}$. This bifurcation is ensured by the transversality condition

$$3\omega_{1}^{4}+2R_{1}\omega_{1}^{2}+R_{2}>0.$$ (16)

**Proof:** Now, considering *λ* as a function of $\tau_{1}$, i.e., $\lambda =\lambda (\tau_{1})$, and differentiating Eq (11) with respect to $\tau_{1}$, we derive the following expression

$$(\frac{d\lambda (\tau_{1})}{d\tau_{1}}{)}^{-1}=-\frac{3\lambda^{2}+2A_{1}\lambda +M_{1}}{\lambda (\lambda^{3}+A_{1}\lambda^{2}+M_{1}\lambda +M_{2})}+\frac{B_{1}}{\lambda (B_{1}\lambda +B_{2})}-\frac{\tau_{1}}{\lambda }.$$ (17)

From this above equation, we get


$$∴\;\text{sign}[Re(\frac{d\lambda (\tau_{1})}{d\tau_{1}}){]}_{\tau_{1}=\tau_{1}^{*}}\;\;=\text{sign}[Re(\frac{d\lambda (\tau_{1})}{d\tau_{1}}{)}^{-1}{]}_{\tau =\tau_{1}^{*},\;\omega =\omega_{1}}\;\;=\text{sign}[3\omega_{1}^{4}+(2M_{1}^{2}-4M_{2}-2B_{1}^{2})\omega_{1}^{2}+(M_{2}^{2}-2M_{1}M_{3}+2B_{1}B_{3}-B_{2}^{2})]\;\;=\text{sign}[3\omega_{1}^{4}+2R_{1}\omega_{1}^{2}+R_{2}].$$

Hence, we conclude that the delayed system (1) undergoes a Hopf bifurcation at the critical value $\tau_{1}=\tau_{1}^{*}$ if the transversality condition $[Re(\frac{d\lambda }{d\tau_{1}}){]}_{\tau_{1}=\tau_{1}^{*}}>0,$ i.e., when $3\omega_{1}^{4}+2R_{1}\omega_{1}^{2}+R_{2}>0$ is satisfied.

**Case - III ($\tau_{1}=0$ and $\tau_{2}>0$)**

In this case, the characteristic Eq (8) reduces to

$$(\lambda +\eta_{S})[(\lambda^{3}+N_{1}\lambda^{2}+N_{2}\lambda +N_{3})+e^{-\lambda \tau_{2}}(C_{1}\lambda^{2}+C_{2}\lambda +C_{3})]=0,\;\text{where}\;\;N_{1}=A_{1}+B_{1},N_{2}=A_{2}+B_{2},\;\text{and}\;N_{3}=A_{3}+B_{3}.$$ (18)

Following a similar approach as for the system described in (10), it can be shown that a critical value $\tau_{2}=\tau_{2}^{*}$ exists for the positive interior equilibrium point *E* , at which the stability transitions for the delayed system (1). Without repeating the detailed analytical calculations presented in **Case - II**, we provide the general expression that characterizes the transition from stability to instability as the value of $\tau_{2}$ increases.

$$\tau_{2}^{n}=\frac{1}{\omega }\cos^{-1}[\frac{(C_{2}-N_{1}C_{1})\omega^{4}+(N_{1}C_{3}+N_{3}C_{1}-C_{2}N_{2})\omega^{2}-C_{3}N_{3}}{C_{1}^{2}\omega^{4}+C_{2}+C_{3}^{2}}]+\frac{2n\pi }{\omega },\;\;\forall n\in \mathbb{N}\cup \{0\}.$$ (19)

Assuming that for $\omega_{2}=\omega (\tau_{2})$, the sequence $\left\{\tau_{2}^{n}\right\}$ represents the critical values of $\tau_{2}$, where n≥0. The minimum value in this sequence, $\tau_{2}^{*}=\min_{n\geq 0}\{\tau_{2}^{n}\}$, is identified as the critical point. The results are summarized in the following theorem without proving it.

**Theorem 3.** *The interior equilibrium E*  *is LAS if* $\tau_{2}<\tau_{2}^{*}$ *and becomes unstable if* $\tau_{2}>\tau_{2}^{*}$, where

$$\tau_{2}^{*}=\frac{1}{\omega_{2}}\arccos[\frac{(C_{2}-N_{1}C_{1})\omega_{2}^{4}+(N_{1}C_{3}+N_{3}C_{1}-C_{2}N_{2})\omega_{2}^{2}-C_{3}N_{3}}{C_{1}^{2}\omega_{2}^{4}+C_{2}+C_{3}^{2}}].$$ (20)

*Moreover, the delayed system (*1*) undergoes a Hopf bifurcation at* $\tau_{2}=\tau_{2}^{*}$ *when transversality condition* $3\omega_{2}^{4}+2R_{1}\omega_{2}^{2}+R_{2}>0$ *hold, where* $R_{1}=M_{1}^{2}-2M_{2}-C_{1}^{2}$ *and* $R_{2}=M_{2}^{2}-2M_{1}M_{3}+2C_{1}C_{3}-C_{2}^{2}$.

**Case - IV ($\tau_{1}>0$ and $\tau_{2}>0$)**

The analysis of this case is extensive and complex, making it challenging to derive precise information regarding the nature of the eigenvalues and the conditions under which stability switches occur. Instead of performing detailed analytical calculations, we have numerically demonstrated the emergence of Hopf-bifurcating periodic solutions for the system (1) and provided the necessary biological interpretations. The result, stated without proof, is presented in the following theorem.

**Theorem 4.** *The non-delayed system is asymptotically stable under the given conditions. However, there exists a critical threshold* $\tau^{*}$ *beyond which the stability of the system undergoes a change. Specifically*

1. *The steady state E*  *remains LAS when* $\tau_{1}+\tau_{2}<\tau^{*}$.
2. *The steady state E*  *becomes unstable when* $\tau_{1}+\tau_{2}>\tau^{*}$.
3. *A Hopf bifurcation occurs at* $\tau_{1}$  +  $\tau_{2}=\tau^{*}$, provided the transversality condition $[Re(\frac{d\lambda }{d\tau }){]}_{\tau =\tau^{*}}>0$ *is satisfied.*

## 4 Optimal control strategy on delayed system

The incorporation of optimal control approaches with delays has become a prominent area of research, particularly due to its applications in biological control systems. In the context of autoimmune responses in psoriasis, the cyclic interactions between T cells and dendritic cells are significantly amplified by the effects of two key cytokines: TNF-*α* and IL-17. This interaction leads to excessive growth of keratinocytes, which is a key characteristic of the disease. To mitigate this, our objective is to determine optimal control strategies for two biologic treatments that minimize the overall treatment cost of psoriasis. We address this by incorporating two control functions, $v_{1}(t)$ and $v_{2}(t)$ into the delayed system (1).

1. $v_{1}(t)$: Represents the effect of a TNF-*α* inhibitor, which can modulate the reaction rates between T cells and dendritic cells.
2. $v_{2}(t)$: Represents the inhibition of the IL-17 axis, targeting its associated pathways.

Thus, the delayed system (1), augmented with optimal control functions, is described by the following set of equations over the specified time interval *[*0,*t*_*f*_*]*.

$$\frac{dT(t)}{dt}=\rho_{T}+\pi T(t)(1-\frac{T(t)}{T^{\max}})-\frac{\mu_{1}\{1-v_{1}(t)\}T(t-\tau_{1})D(t-\tau_{1})}{1+\epsilon_{1}S(t-\tau_{1})}-\delta T(t)K(t)-\eta_{T}T(t),\;\frac{dD(t)}{dt}=\rho_{D}-\frac{\mu_{2}\{1-v_{2}(t)\}T(t-\tau_{2})D(t-\tau_{2})}{1+\epsilon_{2}S(t-\tau_{2})}-\eta_{D}D(t),\;\frac{dK(t)}{dt}=\rho_{K}+\frac{\mu_{1}\{1-v_{1}(t)\}T(t-\tau_{1})D(t-\tau_{1})}{1+\epsilon_{1}S(t-\tau_{1})}+\frac{\mu_{2}\{1-v_{2}(t)\}T(t-\tau_{2})D(t-\tau_{2})}{1+\epsilon_{2}S(t-\tau_{2})}-\eta_{K}K(t),\;\frac{dS(t)}{dt}=\rho_{S}-\eta_{S}S(t),$$ (21)

with $T_{0}(t)>0,\;D_{0}(t)>0,\;K_{0}(t)>0$ and $S_{0}(t)>0,\;\;\forall t\in [-\tau ,0]$.

### 4.1 Description of the objective functional

Our primary objective is to minimize the cost associated with each biologic treatment to reduce the overall treatment expense of psoriasis. To achieve this, we define an objective cost functional for the minimization problem, expressed as follows:

$$J(v_{1}(t),v_{2}(t))=\int_{0}^{t_{f}}[K(t)+L_{1}v_{1}^{2}(t)+L_{2}v_{2}^{2}(t)]dt,$$ (22)

subject to the control system (21). The parameters *L*_1_ and *L*_2_ represent positive weight constants on the benefit of the cost of the TNF-*α* and IL-17 inhibitors, respectively. Our aim is to determine the optimal control $v^{*}(.)=(v_{1}(.),v_{2}(.))$ that satisfies the following:

$$J(v^{*})=\min\{J(v):v(t)\in U_{1}\times U_{2}\}.$$ (23)

The Lebesgue measurable control set is defined on the interval *[*0,*t*_*f*_*]*, where *t*_*f*_ represents the terminal time of control.

$$U_{1}\times U_{2}=\{v(t)=(v_{1},v_{2}):0\leq v_{i}(t)\leq v_{i}^{\max}<1,\forall t\in [0,t_{f}],\;i=1,2\}.$$ (24)

Pontryagin’s Minimum Principle with delays has been applied to establish the necessary conditions for solving this optimal control problem [23]. The optimal control-induced system (21) has been shown to admit non-negative, bounded solutions for a bounded Lebesgue-measurable control function and the non-negative initial conditions.

### 4.2 Optimal control’s characteristics

To characterize the optimal control, Pontryagin’s Minimum Principle reduces the problem (21)–(24) to a problem of minimizing the Hamiltonian *H*, defined as

$$H=K(t)+L_{1}v_{1}^{2}(t)+L_{2}v_{2}^{2}(t)\;+\Gamma_{1}(t)[\rho_{T}+\pi T(t)(1-\frac{T(t)}{T^{\max}})-\frac{\mu_{1}\{1-v_{1}(t)\}T(t-\tau_{1})D(t-\tau_{1})}{1+\epsilon_{1}S(t-\tau_{1})}-\delta T(t)K(t)-\eta_{T}T(t)]\;+\Gamma_{2}(t)[\rho_{D}-\frac{\mu_{2}\{1-v_{2}(t)\}T(t-\tau_{2})D(t-\tau_{2})}{1+\epsilon_{2}S(t-\tau_{2})}-\eta_{D}D(t)]\;+\Gamma_{3}(t)[\rho_{K}+\frac{\mu_{1}\{1-v_{1}(t)\}T(t-\tau_{1})D(t-\tau_{1})}{1+\epsilon_{1}S(t-\tau_{1})}+\frac{\mu_{2}\{1-v_{2}(t)\}T(t-\tau_{2})D(t-\tau_{2})}{1+\epsilon_{2}S(t-\tau_{2})}-\eta_{K}K(t)]\;+\Gamma_{4}(t)[\rho_{S}-\eta_{S}S(t)],$$ (25)

where, $\Gamma_{i}(t)$’s (i=1,2,3,4) are the adjoint variables to be determined suitably. The adjoint system with transversality criteria $\Gamma_{i}(t_{f})=0$ for i=1,2,3,4 can be obtained by using Pontryagin’s Minimum Principle [23] as


$$\frac{d\Gamma_{1}(t)}{dt}=-\frac{\partial H}{\partial T}(t)-\sum_{i=1}^{2}\chi [0,t_{f}-\tau_{i}](t)\frac{\partial H}{\partial T}(t+\tau_{i}),\;\frac{d\Gamma_{2}(t)}{dt}=-\frac{\partial H}{\partial D}(t)-\sum_{i=1}^{2}\chi [0,t_{f}-\tau_{i}](t)\frac{\partial H}{\partial D}(t+\tau_{i}),\;\frac{d\Gamma_{3}(t)}{dt}=-\frac{\partial H}{\partial K}(t),\;\frac{d\Gamma_{4}(t)}{dt}=-\frac{\partial H}{\partial S}(t)-\sum_{i=1}^{2}\chi [0,t_{f}-\tau_{i}](t)\frac{\partial H}{\partial S}(t+\tau_{i}).$$

The optimality of the considered system consists of the optimal states and the corresponding adjoint system takes the form

$$\frac{d\Gamma_{1}(t)}{dt}=-\Gamma_{1}(t)[\pi (1-\frac{2T^{*}(t)}{T^{\max}})-\delta K^{*}(t)-\eta_{T}]\;+\chi [0,t_{f}-\tau_{1}](t)(\Gamma_{1}-\Gamma_{3})(t+\tau_{1})[\frac{\mu_{1}\{1-v_{1}^{*}(t)\}D^{*}(t)}{1+\epsilon_{1}S^{*}(t)}]\;+\chi [0,t_{f}-\tau_{2}](t)(\Gamma_{2}-\Gamma_{3})(t+\tau_{2})[\frac{\mu_{2}\{1-v_{2}^{*}(t)\}D^{*}(t)}{1+\epsilon_{2}S^{*}(t)}],\;\frac{d\Gamma_{2}(t)}{dt}=\eta_{D}\Gamma_{2}(t)+\chi [0,t_{f}-\tau_{1}](t)(\Gamma_{1}-\Gamma_{3})(t+\tau_{1})[\frac{\mu_{1}\{1-v_{1}^{*}(t)\}T^{*}(t)}{1+\epsilon_{1}S^{*}(t)}]\;+\chi [0,t_{f}-\tau_{2}](t)(\Gamma_{2}-\Gamma_{3})(t+\tau_{2})[\frac{\mu_{2}\{1-v_{2}^{*}(t)\}T^{*}(t)}{1+\epsilon_{2}S^{*}(t)}],\;\frac{d\Gamma_{3}(t)}{dt}=-1+\delta T^{*}\Gamma_{1}(t)+\eta_{K}\Gamma_{3}(t),\;\frac{d\Gamma_{4}(t)}{dt}=\eta_{S}\Gamma_{4}(t)-\chi [0,t_{f}-\tau_{1}](t)(\Gamma_{1}-\Gamma_{3})(t+\tau_{1})[\frac{\epsilon_{1}\mu_{1}\{1-v_{1}^{*}(t)\}T^{*}(t)D^{*}(t)}{\{1+\epsilon_{1}S^{*}(t){\}}^{2}}]\;-\chi [0,t_{f}-\tau_{2}](t)(\Gamma_{2}-\Gamma_{3})(t+\tau_{2})[\frac{\epsilon_{2}\mu_{2}\{1-v_{2}^{*}(t)\}T^{*}(t)D^{*}(t)}{\{1+\epsilon_{2}S^{*}(t){\}}^{2}}].$$ (26)

We determine the optimal controls $v_{i}^{*}(t)$ (i=1,2), using the optimality condition provided by

$$\frac{\partial H}{\partial v_{1}}{|}_{v_{1}=v_{1}^{*}(t)}=0\;\text{and}\;\frac{\partial H}{\partial v_{2}}{|}_{v_{2}=v_{2}^{*}(t)}=0.$$ (27)

Taking partial differentiation of the Eq (25) with respect to $v_{1}$ and $v_{2}$ separately and applying the condition (27), we obtain


$$v_{1}^{*}(t)=\frac{1}{2L_{1}}(\Gamma_{3}-\Gamma_{1})(t)[\frac{\mu_{1}T^{*}(t-\tau_{1})D^{*}(t-\tau_{1})}{1+\epsilon_{1}S^{*}(t-\tau_{1})}]=\Pi_{1}(t)\;\;\text{(say)}$$

$$v_{2}^{*}(t)=\frac{1}{2L_{2}}(\Gamma_{3}-\Gamma_{2})(t)[\frac{\mu_{2}T^{*}(t-\tau_{2})D^{*}(t-\tau_{2})}{1+\epsilon_{1}S^{*}(t-\tau_{2})}]=\Pi_{2}(t)\;\;\text{(say)}$$

Now, using the conventional control’s boundedness criteria and following the characteristics of the control set $U_{1}\times U_{2}$ that the admissible control takes the values such that $0\leq v_{i}(t)\leq v_{i}^{\max}<1\;(i=1,2)$, we can have

$$v_{1}^{*}(t)=\left\{ \begin{array}{c} \;\;0,\;\;\;\text{when}\;\Pi_{1}(t)\leq 0\\ \Pi_{1}(t),\;\;\text{when}\;0\leq \Pi_{1}(t)\leq v_{1}^{\max}\\ \;v_{1}^{\max},\;\;\text{when}\;\Pi_{1}(t)\geq v_{1}^{\max} \end{array}\right.\text{and}\;\;v_{2}^{*}(t)=\left\{ \begin{array}{c} \;\;0,\;\;\;\text{when}\;\Pi_{2}(t)\leq 0\\ \Pi_{2}(t),\;\;\text{when}\;0\leq \Pi_{2}(t)\leq v_{2}^{\max}\\ \;v_{2}^{\max},\;\;\text{when}\;\Pi_{2}(t)\geq v_{2}^{\max}. \end{array}\right.$$ (28)

From this result, we can derive the following theorem.

**Theorem 5.** *If the objective cost functional* $J(v_{1}(t),v_{2}(t))$ *attains its minimum value for optimal controls* $v_{1}^{*}(t)$ *and* $v_{2}^{*}(t)$*, moreover (T* , *D* *, K* *, S* *) be the corresponding optimal state for the optimal control problem (*21*), then there exist adjoint functions* $\Gamma_{i}(t)$ *satisfying the transversality conditions* $\Gamma_{i}(t_{f})=0$*, for* i=1,2,3,4*. Furthermore, the optimal control solutions are given by*

$$v_{j}^{*}(t)=\max\{0,\min\{1,\frac{1}{2L_{j}}(\Gamma_{3}-\Gamma_{j})(t)[\frac{\mu_{j}T^{*}(t-\tau_{j})D^{*}(t-\tau_{j})}{1+\epsilon_{j}S^{*}(t-\tau_{j})}]\}\}\;\;\;for,\text{ j=1,2.}$$ (29)

## 5 Numerical simulations

In this section, we perform several numerical simulations to validate our analytical results for both the delayed system (1) and the optimal control system on the delay-induced model (21). The initial values for the model populations are chosen to satisfy the biological assumptions underlying this study. To demonstrate the behavior of the model, numerical simulations have been performed using the parameter values provided in Table 1.

### 5.1 Simulations of the stability of equilibrium

The initial values of all model populations for the simulation are chosen as follows

$$(T(\theta ),D(\theta ),K(\theta ),S(\theta ))=(10,25,140,25),\;\;\theta \in [-\tau ,0],\;\;\text{where}\;\;\tau =\max\{\tau_{1},\tau_{2}\}.$$ (30)

**Fig 2:**
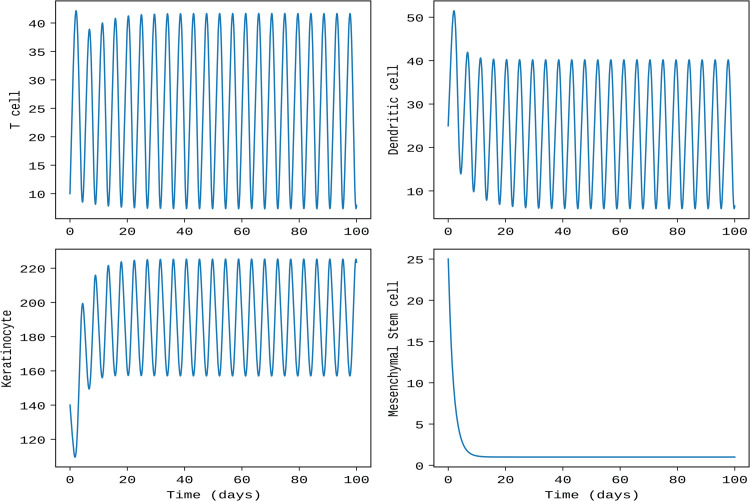
The dynamics of the model populations, namely T cells, dendritic cells, keratinocytes, and mesenchymal stem cells, for the delayed system with $\tau_{1}=\tau_{2}=1.2$.

Initially, when both delay parameters are set to $\tau_{1}=\tau_{2}=0$, representing the non-delayed system, the model populations, namely, T cells [*T*(*t*)], dendritic cells [*D*(*t*)], keratinocytes [*K*(*t*)], and mesenchymal stem cells [*S*(*t*)], exhibit asymptotically stable behavior. However, introducing time delays $\tau_{1}$ and $\tau_{2}$ (see Fig 2), all system populations, except mesenchymal stem cells [*S*(*t*)], show oscillatory dynamics. For this simulation, the intracellular delay parameters are set to $\tau_{1}=\tau_{2}=1.2$, while the remaining parameter values are taken from Table 1.

**Fig 3:**
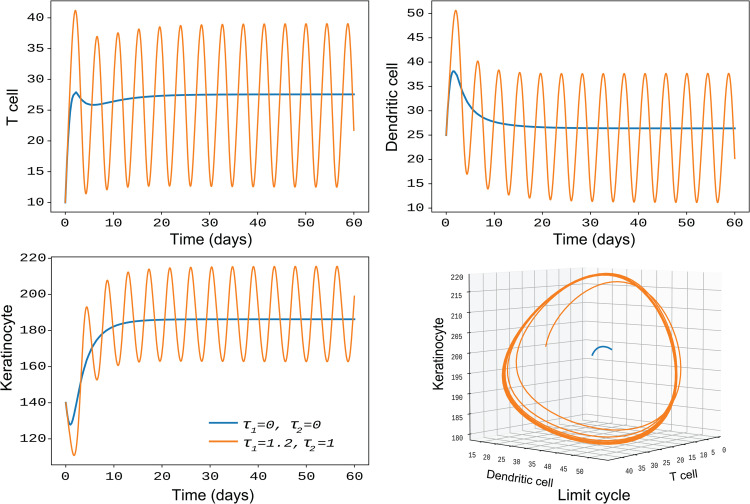
This figure illustrates the populations of three cell types T cells, dendritic cells, and keratinocytes, for both the non-delayed system ($\tau_{1}=\tau_{2}=0$, shown with blue trajectories) and the delayed system ($\tau_{1}=1.2,\tau_{2}=1$, shown with orange trajectories). The introduction of non-zero delays results in periodic solutions, with the corresponding limit cycles depicted in a 3D plot.

Since introducing non-zero delays into the system (1) does not alter the asymptotic behavior of the MSC population, our focus shifts to the dynamics of the other state populations. In (Fig 3), we illustrate the behavior of the first three cell types: T cells, dendritic cells, and keratinocytes, for both non-delayed system ($\tau_{1}=\tau_{2}=0$), represented by blue trajectories and the delayed system ($\tau_{1}=1.2,\tau_{2}=1$), represented by orange trajectories. Without delays, all system populations are asymptotically stable and converge to the endemic steady state. The introduction of non-zero delays exhibits stable periodic oscillations in the populations of these cell types. Moreover, the resulting limit cycles are depicted in a 3-D plot with respect to these populations, providing a comprehensive visualization of the periodic behavior. The simulation demonstrates the significant effect of intracellular delays on the autoimmune response for psoriasis transmission for the considered model system.

### 5.2 Simulations with different time delays

In this subsection, we examine numerically the influence of time delays on first three model populations of the system (1) and perform some simulations in order to depicted the delay induced Hopf bifurcating positions by considering each delay parameters separately at a time.

**Fig 4:**
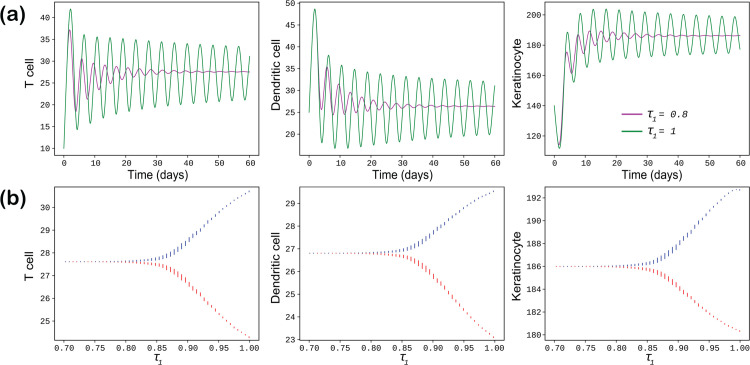
In sub-figure (a), the populations of T cells, dendritic cells, and keratinocytes are shown for the delay parameter $\tau_{2}=0$, with trajectories plotted for two distinct values of $\tau_{1}$ (0.8 and 1), each represented by a different color code. Sub-figure (b) depicts the Hopf bifurcation with respect to the delay parameter $\tau_{1}$ while keeping $\tau_{2}=0$.

If the time delay parameter $\tau_{2}$ is fixed to zero ($\tau_{2}=0$), and $\tau_{1}=0.8$ and $\tau_{1}=1$ respectively, we observe in Fig 4(a) that the oscillation increases as $\tau_{1}$ increases and periodic oscillation is seen at $\tau_{1}=1$ day. For $\tau_{1}=0.8$, the cell densities initially oscillate but eventually achieve local asymptotic stability, confirming our analytical finding stated in **Case-II** in the Sect 3.3. The trajectories for $\tau_{1}=0.8$ and $\tau_{1}=1$ are illustrated using two distinct color codes. In this scenario, the stability of the endemic equilibrium *E*  depends on the value of the time delay $\tau_{1}$, where $\tau_{2}$ is fixed to zero. Larger values of $\tau_{1}$ lead to regular oscillatory solutions, indicating that an increased time delay between the interactions of T cells with dendritic cells and keratinocytes results in sustained oscillations. Stability switch occurs through Hopf bifurcation which are depicted in the Fig 4(b) where we present the delay-induced Hopf-bifurcation, focusing on T cells, dendritic cells, and keratinocyte cells populations only. In this analysis, $\tau_{1}$ serves as the bifurcation parameter. The parameter $\tau_{1}$ is varied within the range of 0.7 to 1. The figure illustrates that, for $\tau_{2}=0$, there exists a critical value $\tau_{1}^{*}$ at which the endemic equilibrium point *E*  transitions from stability to instability. This observation highlights the critical role of the delay parameter $\tau_{1}$ in determining the stability of the system (1). Biologically, this implies that an increase in the time lag within the interaction term of the T cell growth equation—mediated by dendritic cell mediated cytokines infiltrating and accelerates fluctuations in the model cell concentrations, excluding the MSC population. These fluctuations manifest as oscillatory dynamics and this behavior arises from the inflammatory response in the upper epidermal layer caused by the over-expression of keratinocytes, which, in turn, amplifies the progression and dissemination of the disease.

**Fig 5:**
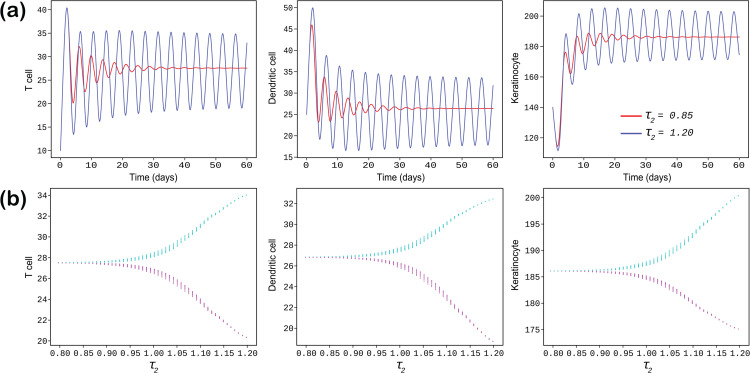
In Sub-figure (a), the populations of T cells, dendritic cells, and keratinocytes are shown with a fixed delay of $\tau_{1}=0$. The trajectories are plotted for two distinct values of $\tau_{2}$ (0.85 and 1.2), each represented by a different color. Sub-figure (b) illustrates the Hopf bifurcation with respect to the delay parameter $\tau_{2}$, while the other delay parameter, $\tau_{1}$, is fixed at 0.

In Fig 5(a), the time delay $\tau_{1}=0$ is fixed, and the trajectories are plotted for three distinct values of $\tau_{2}$, color coded as follows: red for $\tau_{2}=0.85$, and blue for $\tau_{2}=1.2$. It is evident that as the value of $\tau_{2}$ increases, the oscillations in the system populations become more pronounced initially but gradually diminish over time, indicating stabilization of the system. This demonstrates that larger values of $\tau_{2}$ contribute to enhanced system stability. In Fig 5(b), we present the bifurcation diagram by varying the bifurcation parameter $\tau_{2}$ from 0.8 to 1.2 while keeping $\tau_{1}=0$. The diagram reveals the occurrence of a Hopf bifurcation at a critical value $\tau_{2}=\tau_{2}^{*}$. For $\tau_{2}<\tau_{2}^{*}$, all system populations except the mesenchymal stem cell (MSC) exhibit stable behavior, whereas for $\tau_{2}>\tau_{2}^{*}$, the stability is lost. This indicates that increasing the time delay in the interaction term of the dendritic cell growth equation (stimulated by T cell-derived cytokines) infiltrating the keratinocyte population induces more oscillatory behavior in the system, excluding the MSC population. This observation highlights the significant impact of the bifurcation parameter $\tau_{2}$ on system stability from a biological point of view.

**Fig 6:**
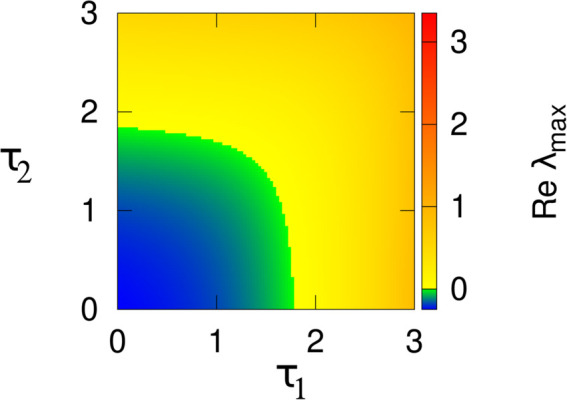
The stability region of the endemic equilibrium point is shown in the $\tau_{1}-\tau_{2}$ plane. The color coding denotes the max[Re(λ)], where *λ* is the eigenvalues corresponding to the delayed system.

In Fig 6, the stability region of the endemic equilibrium point is depicted in the $\tau_{1}$ − $\tau_{2}$ plane. The color gradient represents the maximum real part of the eigenvalue, denoted as $Re(\lambda_{\max})$, where *λ* corresponds to the eigenvalue of the Jacobian matrix at the interior equilibrium point of the delayed system (1). The equilibrium point is unstable in the region where $Re(\lambda_{\max})>0$. It is observed that the critical values of $\tau_{1}$ and $\tau_{2}$, denoted as $\tau_{1}^{*}$ and $\tau_{2}^{*}$, increase in the figures. Consequently, the area of the stability region for the endemic equilibrium expands with increasing values of $\tau_{1}$ and $\tau_{2}$. The values of all other parameters used in the analysis are provided in Table 1.

### 5.3 Simulations of optimal control for the delayed model

In this subsection, we numerically solve the optimal control problem for the delayed system (21) and present the results obtained through simulation. The parameter values used for the simulations are taken from Table 1, while the initial values are specified in Eq (30). The weight constants in the objective functional are set as $L_{1}=L_{2}=0.5$.

The optimal system (21) represents a two-point boundary value problem (BVP) with boundary conditions provided at two time points, *t* = 0 and *t* = *t*_*f*_. For the numerical simulations, we utilized the **bvp4c** solver in **MATLAB**, which is specifically designed for solving nonlinear two-point BVPs. For a detailed understanding of this numerical method, readers may refer to the work by Torres et al. [26].

**Fig 7:**
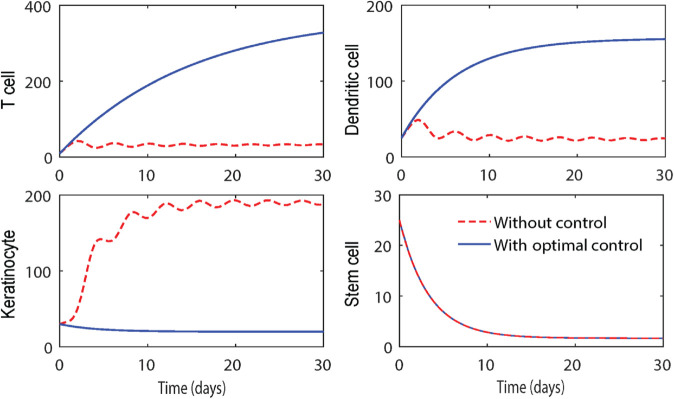
Numerical solution of the optimal control problem with $\tau_{1}=\tau_{2}=1.1$ and remaining parameter values taken from Table 1. The red dotted trajectories represent the disease dynamics without optimal control, while the blue trajectories depict the population behavior under the influence of optimal control.

To solve the system, we define the state variables *T*(*t*), *D*(*t*), *K*(*t*), *S*(*t*), the adjoint variables $\Gamma_{1}(t),\Gamma_{2}(t),\Gamma_{3}(t),$ $\Gamma_{4}(t)$, and the control functions $\left(v_{1}(t),v_{2}(t)\right)$. A combination of forward-backward difference approximations is employed to solve the control-induced system along with the adjoint system. The solutions are then plotted, with all state variables shown over time in Fig 7. The red dotted trajectories represent the system population concentrations without control, while the blue trajectories correspond to the population behavior under optimal control.

**Fig 8:**
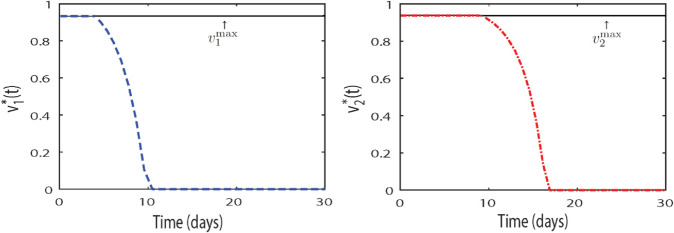
Numerical approximation of Pontryagin’s extremals and the corresponding optimal control profiles $v_{1}^{*}(t)$ and $v_{2}^{*}(t)$.

To determine the optimal control profiles, we apply the transversality conditions and enforce the boundedness of controls within the interval [0,1] as per Pontryagin’s principle. The control functions $v_{1}^{*}(t)$ and $v_{2}^{*}(t)$ for the delayed system (21) are computed and plotted over 30 days, as shown in Fig 8. From the results, we observe a similar pattern for both control profiles, though notable differences exist. The control $v_{1}^{*}$ (represented by trajectories with blue dots), associated with the TNF-*α* inhibitor drug, modulates the system within a short span of approximately 10 days. In contrast, the control $v_{2}^{*}$ (indicated by red dotted trajectories), corresponding to the IL-17 inhibitor, requires about 18 days to regulate the system effectively. This indicates that the combination of these biologic inhibitors optimally controls the delayed model system, mitigating the auto-immune response and keratinocyte over-expression. The inflammatory cytokine loop, regulated predominantly by T cell and dendritic cell mediated interactions, is thus brought under control, achieving an optimal therapeutic outcome.

**Fig 9:**
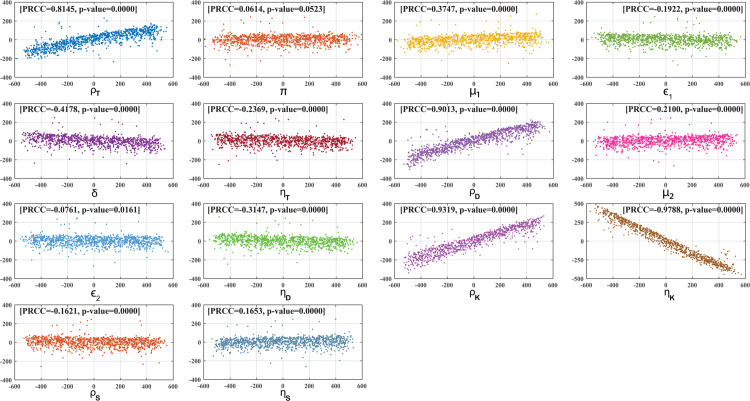
PRCC scatter plots for all system parameters against the keratinocyte population, with a sample size of N = 1000. The PRCC values are calculated at 100 days and all p values are taken as up to four decimal places. The x-axis shows the residuals obtained from regressing the rank-transformed values of the parameter of interest on the rank-transformed values of all other parameters. The y-axis displays the residuals from regressing the rank-transformed keratinocyte values on the rank-transformed values of all other parameters, excluding the one being analyzed.

In addition to these, Fig 9 presents the global sensitivity analysis of all parameters in our system using Latin Hypercube Sampling (LHS) with the Partial Rank Correlation Coefficients (PRCC) method, focusing on the keratinocyte population. We evaluate the PRCC and p-value for each parameter, where the p-value indicates the level of uncertainty associated with each parameter’s PRCC value. p-value <0.05 for each parameter signifies the statistical significance of the corresponding PRCC value. This scatter plot shows that the parameters $\rho_{T}$, $\mu_{1}$, $\rho_{D}$, and $\rho_{K}$ have a positive influence on disease progression, while the parameters *δ*, $\eta_{T}$, $\eta_{D}$, and $\eta_{K}$ have a negative influence. In this study, we consider the keratinocyte level as a marker for disease prediction. The overall sensitivity analysis helps in identifying the precise point of treatment for psoriatic disease.

## 6 Discussion and conclusion

Autoimmune diseases like psoriasis involve complex immune mechanisms, where time delays play a crucial role in the onset and progression of symptoms. These delays arise from processes such as immune activation, cytokine production, and cellular recruitment. Specifically, the formation of synergistic loops by pro-inflammatory cytokines and the subsequent regulatory response by anti-inflammatory cytokines are inherently non-instantaneous. Discrete intracellular delays represent measurable periods between the triggering event and disease manifestation, varying based on patient characteristics, environmental factors, and the specific autoimmune condition. For psoriatic lesions, while symptoms may appear soon after a trigger, the full extent of the disease evolves over a longer time-frame.

In this study, we incorporated two intracellular immune response delays, $\tau_{1}$ and $\tau_{2}$, into our mathematical model to represent the temporal dynamics of immune interactions in psoriasis. The first delay, $\tau_{1}$, reflects the time required for the initial immune response, such as T cell activation and proliferation triggered by dendritic cells. The second delay, $\tau_{2}$, accounts for subsequent processes, including cytokine production by activated T cells and the recruitment of additional immune cells to the site of inflammation.

The bifurcation analysis revealed the critical role of these delays in the system’s stability. When $\tau_{1}$ is fixed and $\tau_{2}$ is varied, we identified a critical threshold $\tau_{2}^{*}$ at which the system transitions from stability to instability. This suggests that the second delay, $\tau_{2}$, significantly influences the overall immune response. Similarly, varying $\tau_{1}$ while keeping $\tau_{2}$ fixed revealed a critical value $\tau_{1}^{*}$, beyond which the system loses stability. These findings underscore the importance of both delays in regulating the immune response and maintaining equilibrium in the system.

The incorporation of two distinct delays provides a realistic representation of the temporal dynamics of psoriasis-related immune responses. Our results highlight that both $\tau_{1}$ and $\tau_{2}$ critically affect the stability of the endemic equilibrium point *E* . Beyond certain threshold values, these delays can induce oscillatory behavior in the system, highlighting the destabilizing effects of prolonged immune response delays. This phenomenon has been identified through the stability region plot presented in the numerical simulation section.

Furthermore, we implemented optimal controls on the delayed model by incorporating the effects of two biologic inhibitors targeting the TNF-*α* and IL-17 pathways. These effects are incorporated into the model with associated cost functions to reduce their high therapeutic expense. Using Pontryagin’s Minimum Principle, we analytically and numerically derived the optimality conditions for the delayed system. The results indicate that the optimal control profiles for the biologics effectively regulate the dynamics of the system, except for the last population, by reducing oscillations and stabilizing the immune response. Notably, the TNF-*α* inhibitor demonstrated faster modulation, achieving control within approximately 10 days, while the IL-17 inhibitor required a longer duration of 18–20 days to achieve effective regulation.

The results of this study, particularly the analysis of delay effects, have important implications for clinical strategies in managing psoriasis. Our findings indicate that therapeutic interventions targeting the timing of immune responses can play a key role in disease control. Specifically, addressing cytokine signaling delays and stabilizing immune responses may help to reduce the chronic inflammation typically observed in psoriasis. The use of delay differential equations in the control-induced model enables clinicians and researchers to better understand and predict the timing of keratinocyte proliferation and immune cell interactions. In addition, the incorporation of optimal control provides a cost-effective approach to biologic therapy, which may help to lower the economic burden of long-term treatment. This study highlights the importance of including temporal dynamics in therapeutic models, thereby offering new insights for the design of advanced treatment strategies for psoriasis. In practice, this could support the development of optimized treatment protocols—such as adjusting the timing of immunosuppressive drug delivery—to minimize flare-ups and improve patient outcomes. Finally, the insights into delay-induced instability also support personalized medicine approaches, where treatment schedules can be adapted to the specific response profile of individual patients.
